# A case report of hemosuccus pancreaticus: the cause of upper gastrointestinal bleeding demystified after 9 months of episodic bleeding

**DOI:** 10.3389/fgstr.2024.1433278

**Published:** 2024-11-20

**Authors:** Marie Solange Mukanumviye, Dyna Nyampinga, Zainab Ingabire, Cedric Kwitonda, Felicien Shikama, Eric Rutaganda, Berhane Redae, Hanna Aberra, Marcellin Musabende, Peter Crook, Jean de Dieu Mbanzabugabo, Ferehiwot Bekele Getaneh, Jean Jacques Nshizirungu

**Affiliations:** ^1^ Fellowship in Gastroenterology & Hepatology, King Faisal Hospital, University of Rwanda, Kigali, Rwanda; ^2^ Head of Endoscopy Unit, Department of Internal Medicine, Kigali University Teaching Hospital, Kigali, Rwanda; ^3^ Department of Gastroenterology, College of Medicine and Health Sciences, King Faisal Hospital, University of Rwanda, Kigali, Rwanda; ^4^ Department of Internal Medicine, Butare University Teaching Hospital, Kigali, Rwanda; ^5^ Visiting Faculty/King Faisal Hospital, Institute for Infection & Immunity, St George’s, University of London, London, United Kingdom; ^6^ Radiology, University of Rwanda, Kigali, Rwanda; ^7^ Department of Radiology, King Faisal Hospital, Kigali, Rwanda

**Keywords:** hemosuccus pancreaticus, pancreatitis, gastroduodenal artery pseudoaneurysm, Rwanda, case report

## Abstract

Hemosuccus pancreaticus is a rare but potentially fatal cause of upper gastrointestinal (GI) bleeding. It is defined as bleeding from the pancreatic duct with blood draining into the duodenum through the ampulla of Vater. In patients with pancreatitis, peri-pancreatic blood vessels may be inflamed by pancreatic enzymes and form a pseudoaneurysm which can rupture and bleed into the pancreatic duct. We report a case of a 43-year-old man who presented with episodic upper GI bleeding of unclear etiology over 9 months without a clear documented history of pancreatitis. The etiology remained elusive even after multiple upper and lower endoscopies. Computed tomography angiography of the abdomen and pelvis during an acute episode detected a pseudoaneurysm of the gastroduodenal artery (GDA) with contrast extravasation into the dilated pancreatic duct. The pseudoaneurysm was treated with coil embolization, resulting in a persisting resolution of the patient’s symptoms. Clinicians should consider abdominal angiography when diagnosing obscure GI bleeding.

## Introduction

Hemosuccus pancreaticus refers to bleeding from the pancreatic duct with blood draining into the duodenum through the ampulla of Vater. It is a rare but potentially fatal cause of upper gastrointestinal (GI) bleeding. It represents 1/1500 cases of upper GI bleeding and it is more common in men aged 50-60 years with a male-to-female ratio of 7:1. Mortality is very high at 90% in the absence of treatment and is approximately 27-37% with treatment (1, 2). Bleeding from the ampulla of Vater was first described by Lower and Farell in 1931. It was later named hemosuccus pancreaticus by Philip Sandholm in 1970 after he identified three patients with pseudoaneurysms that ruptured into the pancreatic duct (2). We present this case to raise awareness of this challenging condition and fatal cause of upper GI bleeding. This case of hemosuccus pancreaticus is illustrated by the abdominal computed tomography angiography (CTA) done during episodes of active bleeding that we present here with contrast extravasation into the pancreatic duct from the ruptured pseudoaneurysm. We also present a general review of this rare but important condition.

## Case presentation

A 43-year-old man was referred for evaluation of upper GI bleeding with unclear etiology after undergoing unremarkable upper and lower GI endoscopies. He reported 9 months of intermittent hematemesis and rectal bleeding, including both fresh blood from the rectum and melena. Each episode of bleeding was preceded by abdominal fullness and severe epigastric pain for approximately 5 hours. He denied experiencing fever, weight loss, night sweats, diarrhea, abdominal distension, or swelling of the lower limbs. For 1 month, the bleeding became so frequent that it required hospitalization with a transfusion of a total of 17 units of packed red blood cells (PRBCs) throughout the 30-day admission. The most recent bleeding episode was the night before his transfer. He had no family history of gastrointestinal malignancy. He used to consume alcohol, two to three drinks on alternate days but had stopped since the beginning of the 9-month illness. He did not have a documented history of acute pancreatitis but reported a history of severe epigastric pain managed as dyspepsia at a health center where there are no facilities to diagnose acute pancreatitis. He tested negative for hepatitis B, hepatitis C, and HIV. He was from an urban village in Rwanda and worked as a businessman. He had no significant freshwater exposure to suggest a risk of schistosomiasis.

At admission, he was weak and brought in a wheelchair. He was oriented and pale. He was tachycardic but normotensive (heart rate 123 beats per minute, blood pressure 110/65 mmHg). He was afebrile with a normal respiratory rate and oxygen saturation on room air. On gastrointestinal examination, there was no jaundice, gynecomastia, hair loss, or spider naevi. He had no abdominal distension or collaterals but there was mild epigastric tenderness. Rectal, respiratory, and cardiovascular examinations were also normal.

His admission hemoglobin (Hb) was 4.6 g/dL, rising to 8.8 g/dL after 3 units of PRBCs. His white blood cell (WBC) count was 11.24 x10^9^/L with an absolute neutrophil count of 8.18 x10^9^/L. Platelets were mildly reduced at 117 x10^9^/L, as were albumin (26.3 g/L) and total protein (43.3 g/L). Amylase and lipase were marginally elevated (159.6 and 125 IU/L respectively). Other work-ups including international internalized ratio (INR), alkaline phosphatase, gamma- glutamyl transferase, alanine transaminase, aspartate aminotransferase, urea, creatinine, potassium, sodium, and C-reactive protein (CRP) were all normal.

On the night of admission, the patient developed hypoxia with oxygen saturation of 86% on room air and a fever of 38.6°C. A chest x-ray found fluid in the right upper fissure and infiltrates in the right lower zone. Given his long-lasting previous hospital stay, he was treated with meropenem for suspected hospital-acquired pneumonia. On day 2 of admission, urgent esophagogastroduodenoscopy (EGD) did not reveal any source of bleeding. The mucosa of the upper GI tract looked normal with no evidence of blood in the upper GI tract. A colonoscopy was conducted up to the terminal ileum without a source of bleeding being identified.

While undergoing CTA of the abdomen and pelvis on day 3 of admission, the patient felt severe abdominal pain and fullness again. He became hypotensive and pale. He was rushed to the high dependency unit (HDU) for an emergency transfusion. In total, four units of PRBCs, four units of platelets, and four units of fresh frozen plasma were administered.

**Figure 1:**
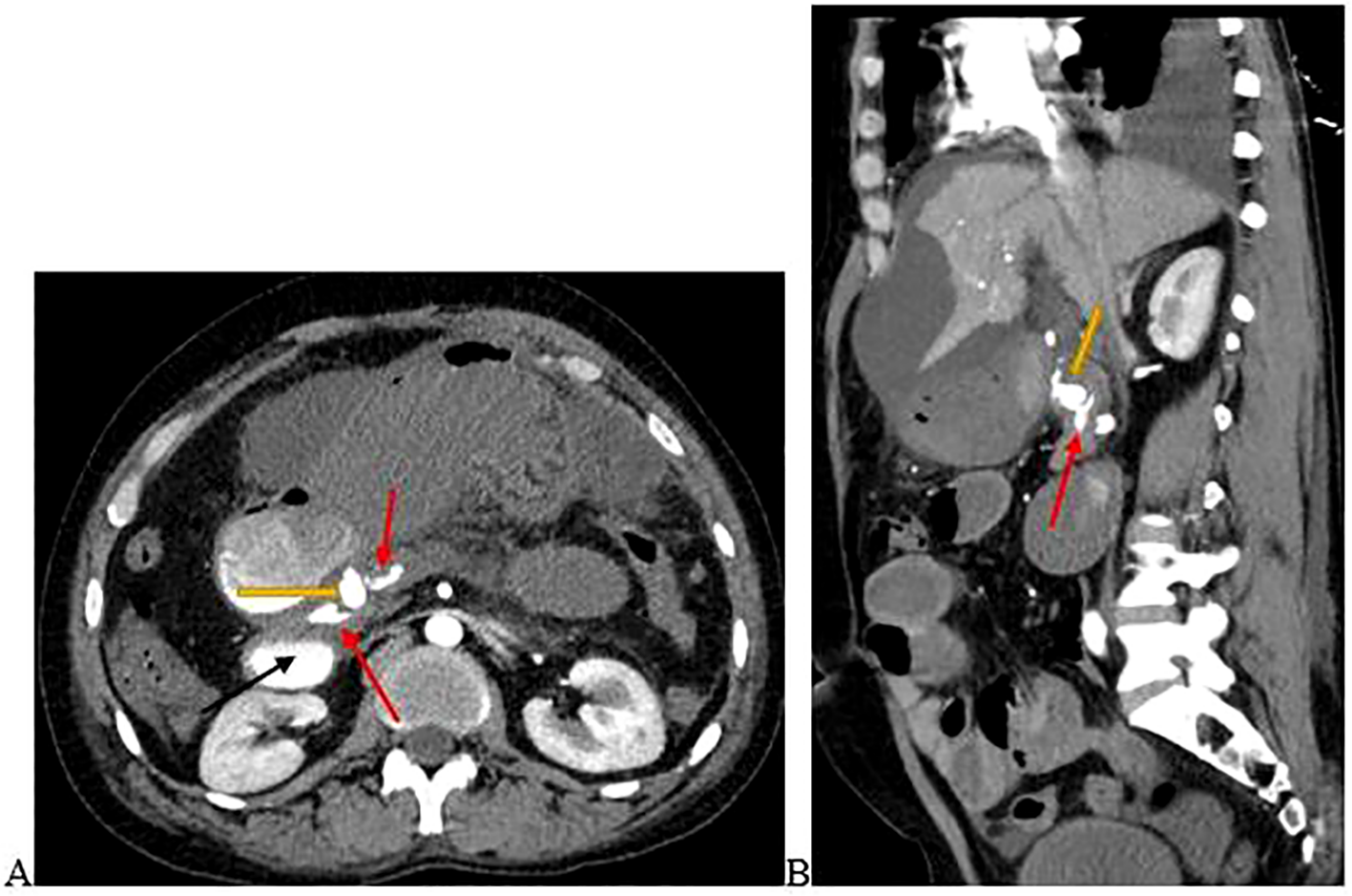
**(A)** is the axial view and **(B)** is the sagittal view of the abdominal computed tomography angiography scan demonstrating a pseudoaneurysm arising from the GDA (yellow arrow) that had ruptured into the pancreatic duct. Contrast extravasation into the pancreatic duct (red arrows) and duodenum (black arrow) occurred. Images were taken on day 3 of admission.

The CTA revealed a pseudoaneurysm arising from the gastroduodenal artery (GDA) that had ruptured into the pancreatic duct. Features of chronic pancreatitis were demonstrated with contrast extravasation within the dilated pancreatic duct. There was also contrast pooling in the duodenum, the rest of the small bowel, and the stomach (**Figure 1**).

The patient was diagnosed with recurrent upper GI bleeding due to hemosuccus pancreaticus as a consequence of a ruptured pseudoaneurysm of the GDA into the pancreatic duct.

After resuscitation, an interventional radiologist (IR) performed coil embolization of the pseudoaneurysm. Under sterile conditions, with access via the right femoral artery, a guide wire was inserted into the gastro-duodenal artery-gastroepiploic artery (GDA-GEA) under fluoroscopic guidance. A 5F sheath was inserted into the GDA-GEA and pseudoaneurysm. Finally, coils were inserted in the GEA with gel-foam and the sheath was removed. After the procedure, the patient was kept in the HDU for close monitoring.

### Follow-up

**Figure 2:**
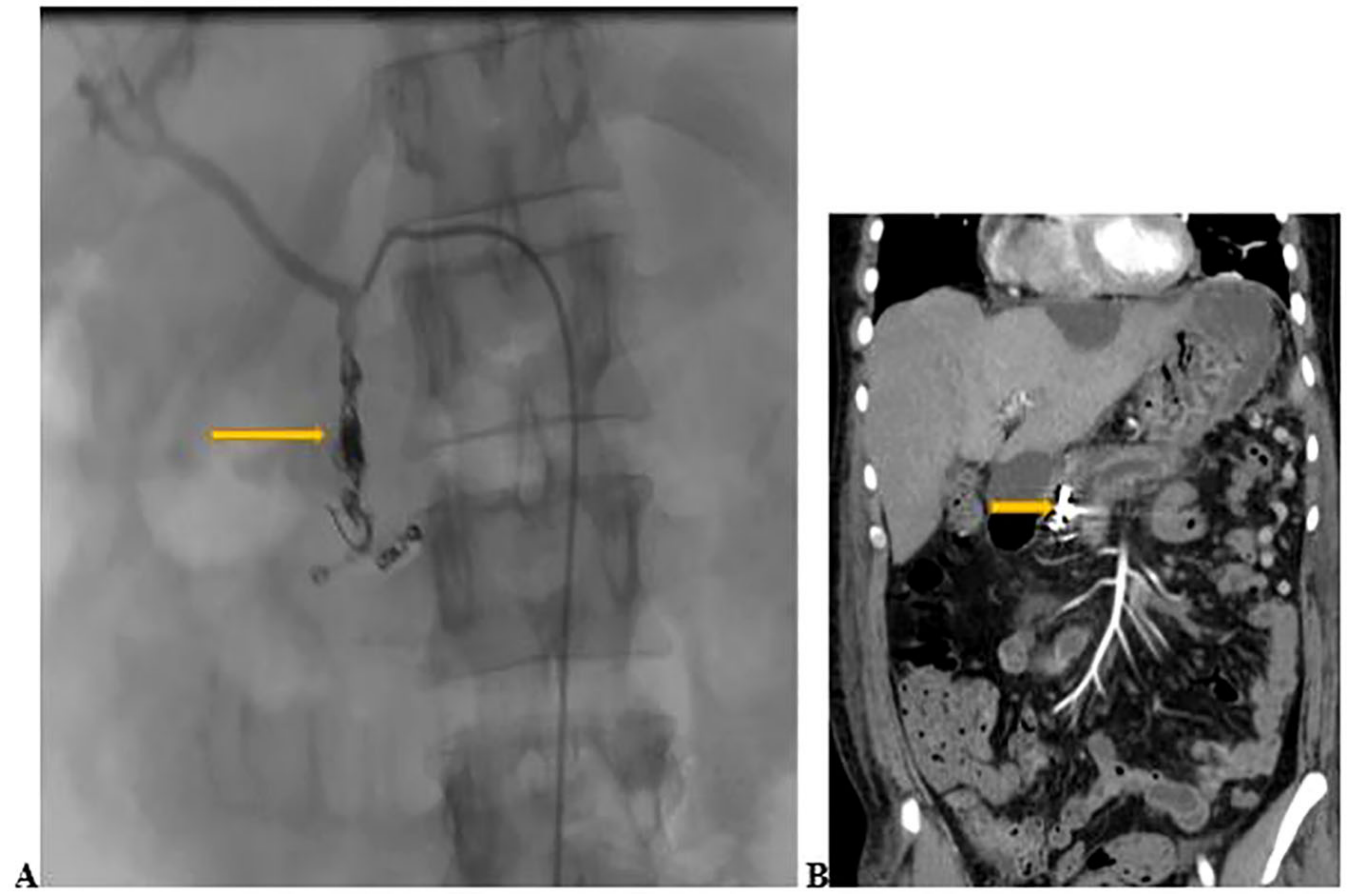
**(A)** Coil and gel-foam embolization of the GDA (yellow arrow) done on day 3 of admission. **(B)** Contrast- enhanced computed tomography scan of the abdomen conducted 2 days after embolization showing embolization of the GDA (yellow arrow) without active bleeding.

After 24 hours of uneventful monitoring in the HDU, the patient was transferred to the medical ward. On the post-procedure contrast-enhanced computed tomography (CECT) scan, the embolization of the GDA was demonstrated without evidence of contrast extravasation (bleeding) (**Figure 2**). He completed 7 days of meropenem for presumed pneumonia. He was weaned from oxygen, and discharged on iron supplements. On review 4 weeks later, he denied any further hematemesis or hematochezia. His repeat hemoglobin showed an increase to 11.3 g/dl. An elective splenectomy was planned to prevent the development of gastric varices as a complication of his chronic splenic vein thrombosis.

## Discussion

In patients with pancreatitis, peri-pancreatic blood vessels may be digested by pancreatic enzymes. The affected blood vessels form a pseudoaneurysm which can rupture at any time, bleed into the pancreatic duct, and cause a fatal condition known as hemosuccus pancreaticus. In our case, hemosuccus pancreaticus was due to a pseudoaneurysm of the GDA. Other causes of hemosuccus pancreaticus include a pancreatic tumor, pancreatic divisum, vascular malformations, iatrogenic injury (post-endoscopic ultrasound and final needle aspiration), or accidental trauma (2). For patients who present with upper GI bleeding with an unidentified source after EGD, a history of pancreatitis should indicate the possibility of bleeding from the ampulla of Vater (1).

In hemosuccus pancreaticus, the bleeding source can be from the splenic, gastroduodenal, pancreaticoduodenal, gastric, or hepatic arteries (with a respective prevalence of 40%, 30%, 20%, 5%, and 2%). The rupture of the pseudoaneurysm may cause bleeding into the luminal gastrointestinal system, a pancreatic pseudocyst, the peritoneal cavity, or pancreatic tissue (3–6). Unlike bleeding from a pancreatic abscess or a stone invading the pancreatic parenchyma, the bleeding from hemosuccus pancreaticus is episodic and most patients retain hemodynamic stability. Sometimes, however, the bleeding is severe and patients can present in shock (7). Our patient suffered intermittent hematemesis with both melena and hematochezia. The bleeding induced hemodynamic instability which required transfusions.

Hemosuccus pancreaticus poses diagnostic challenges due to its rarity, anatomical location, and intermittent bleeding (3, 8, 9). It is hard to diagnose as the patient may not have obvious bleeding at the time of examination. Laboratory investigations may be unrevealing, although bilirubin and pancreatic enzymes can be elevated. Abdominal imaging is helpful to confirm the diagnosis. Imaging modalities to diagnose this condition include EGD (helpful to exclude the more common causes of upper GI bleeding), abdominal ultrasound, abdominal CT scan with contrast, abdominal angiography, endoscopic ultrasound, and endoscopic retrograde cholangiopancreatography (2).

Among the diagnostic modalities, abdominal CTA is the gold standard as it can detect the aneurysm in 90% of cases and may identify calcifications suggestive of chronic pancreatitis (2), but requires a high bleeding rate to detect the bleeding vessels (10). In cases in which EGD is conducted during active bleeding, the endoscopist may see blood coming from the ampullar of Vater (2). In rare cases, the diagnosis remains elusive, and an explorative laparotomy can be performed (1). For our case report, EGD failed to detect the bleeding because of the anatomical location of the source of bleeding (bleeding from the pancreatic duct) and we could not see the bleeding from the ampulla of Vater as the patient was not actively bleeding during the time of the endoscopy. Abdominal CTA detected the bleeding as the patient had active bleeding while undergoing the investigation.

The first line treatment of hemosuccus pancreaticus is arterial embolization. Surgery is an alternative for patients for whom embolization fails or the diagnosis remains unclear after exhaustive investigations (11). A retrospective Indian study reported 31 cases of hemosuccus pancreaticus from 1997 to 2008 and 50% of the patients had successful embolization. Surgery was performed for urgent cases and in those for whom arterial embolization failed. The surgical options that were used were distal pancreatectomy and splenectomy in 11 patients, central pancreatectomy in 2 patients, intracystic ligation of the blood vessel for 6 patients, and aneurysmal ligation and bypass graft for one patient (7).

## Conclusion

Hemosuccus pancreaticus is a rare but potentially fatal cause of upper GI bleeding. It is characterized by intermittent upper GI bleeding, often with a background of pancreatitis. Clinicians should suspect it in cases of upper GI bleeding with normal EGD and a past history of pancreatic diseases. The diagnosis is best made by abdominal CTA during active bleeding. The prognosis of the disease has become better with the availability of interventional radiologists who perform arterial embolizations.

## Patient perspective

This case report presents a 43-year-old man with a rare cause of upper GI bleeding, hemosuccus pancreaticus. While the clinical aspects of this case provide critical insights into the disease diagnosis and treatment, understanding the patient’s personal experience adds a valuable dimension to the discussion. The patient’s main symptoms were massive hematemesis, followed by rectal bleeding and melena. Before the onset of these, he noticed severe epigastric pain. He remembered that one of the doctors told him that the investigation was in favor of pancreatitis, but the origin of the bleeding was mysterious before meeting the expert doctors from our institution. He even thought that it was a punishment from God or poison. The bleeding was frequent, requiring him to stay in the hospital for repetitive transfusions in order to survive. He was unable to carry out his usual activities. After fixing his problems, he was back to his usual job and he is enjoying life with his family of four kids and wife. He is very grateful for the great job we have done for him to save his life. He is very proud of disseminating this information as this will help in raising awareness of the disease and its management. This knowledge may help patients with the same condition and features.

## Data Availability

The original contributions presented in the study are included in the article/supplementary material. Further inquiries can be directed to the corresponding author.
